# MXRA8 is an immune-relative prognostic biomarker associated with metastasis and CD8^+^ T cell infiltration in colorectal cancer

**DOI:** 10.3389/fonc.2022.1094612

**Published:** 2023-01-10

**Authors:** Lulu Tan, Daan Fu, Feng Liu, Jia Liu, Yang Zhang, Xin Li, Jinbo Gao, Kaixiong Tao, Guobin Wang, Lin Wang, Zheng Wang

**Affiliations:** ^1^ Department of Gastrointestinal Surgery, Union Hospital, Tongji Medical College, Huazhong University of Science and Technology, Wuhan, China; ^2^ Department of Anesthesiology, Union Hospital, Tongji Medical College, Huazhong University of Science and Technology, Wuhan, China; ^3^ Research Center for Tissue Engineering and Regenerative Medicine, Union Hospital, Tongji Medical College, Huazhong University of Science and Technology, Wuhan, China; ^4^ Department of Clinical Laboratory, Union Hospital, Tongji Medical College, Huazhong University of Science and Technology, Wuhan, China

**Keywords:** MXRA8, metastasis, CD8^+^ T cell, immune infiltration, colorectal cancer

## Abstract

**Background:**

Colorectal cancer (CRC) is the second most common cause of cancer-related deaths worldwide. Tumor metastasis and CD8^+^ T cell infiltration play a crucial role in CRC patient survival. It is important to determine the etiology and mechanism of the malignant progression of CRC to develop more effective treatment strategies.

**Methods:**

We conducted weighted gene co‐expression network analysis (WGCNA) to explore vital modules of tumor metastasis and CD8^+^ T cell infiltration, then with hub gene selection and survival analysis. Multi-omics analysis is used to explore the expression pattern, immunity, and prognostic effect of MXRA8. The molecular and immune characteristics of MXRA8 are analyzed in independent cohorts, clinical specimens, and *in vitro.*

**Results:**

MXRA8 expression was strongly correlated with tumor malignancy, metastasis, recurrence, and immunosuppressive microenvironment. Furthermore, MXRA8 expression predicts poor prognosis and is an independent prognostic factor for OS in CRC.

**Conclusion:**

MXRA8 may be a potential immunotherapeutic and prognostic biomarker for CRC.

## Introduction

Colorectal cancer (CRC) is the third most common cancer and the second most common cause of cancer-related deaths worldwide (1). Approximately 20% of CRC patients have been reported to have progressed to a metastatic state at presentation, and up to 50% of localized CRC patients eventually present with metastatic disease (2, 3). The prognosis of metastatic CRC (mCRC) patients remains poor, with a three-year survival rate of less than 30% (4). Therefore, it is important to determine the etiology and mechanism of the malignant progression of CRC to develop more effective treatment strategies.

Immunotherapy, especially immune checkpoint inhibitors (ICIs), has become one of the effective therapeutic options for mCRC (5). ICIs have shown promising success in non-small cell lung cancer, metastatic melanoma, metastatic bladder cancer and prostate cancer (6, 7). However, ICIs demonstrated very limited clinical activity in mCRC. An important molecular mechanism of ICIs resistance is insufficient CD8^+^ T cell infiltration or loss of CD8^+^ T cell function (8). Studies have shown that the extent and activity of CD8^+^ T cells can affect tumor prognosis and immunotherapy response rates (9, 10). Less infiltration of CD8^+^ T cells in the center of tumor focus, has restricted the efficiency of immunotherapy in CRC (11). Therefore, identifying biomarkers and mechanisms of reduced infiltration and dysfunction of CD8^+^ T cells in CRC is critical for mCRC immunotherapy.

This study explored potential prognostic biomarkers and their biological functions in CRC, identifying matrix remodeling associated protein 8 (MXRA8) as a target gene. MXRA8 is a receptor for various articular viruses (12), but its role in cancer development and progression remains unsolved. Studies have demonstrated that MXRA8 is highly expressed in most solid tumor tissues compared to adjacent normal tumors (13), and it can modulate iron death and promote glioma progression (14). High MXRA8 expression is associated with poorer overall survival in clear cell renal cell carcinoma (15), but the potential function of MXRA8 in CRC has not been elucidated. In current study, highly expressed of MXRA8 was first determined in CRC tissues, and verified to promote invasion and metastasis in CRC cell. Furthermore, the expression level of MXRA8 reflects abnormal immune status in CRC, including infiltration and dysfunction of CD8^+^ T cells. Therefore, MXRA8 can be used a potential immunotherapeutic and prognostic biomarker for CRC.

## Materials and methods

### Data preprocessing

The expression profile of CRC tissues in GSE87211, GSE39582, GSE38832, GSE16158 and GSE16537 datasets were downloaded from GEO database (http://www.ncbi.nlm.nih.gov/geo/). GSE87211 dataset was used for module and gene selection significantly associated with CRC metastasis and weighted gene co-expression networks analysis (WGCNA) establishment. GSE39582 dataset was used as the training cohort to construct the prognostic prediction model. TCGA-COAD normalized data and clinical information were downloaded from UCSC Xena website (https://xenabrowser.net). GSE38832, GSE16158 and GSE16537 dataset were used as validation cohort. All gene expression profiles were normalized by R software.

### Weighted gene co-expression networks analysis

The top 25% of genes with the largest variance in GSE87211 were selected for further co-expression network construction. To ensure the reliability of the results, an outlier was removed. Module identification was accomplished with the dynamic tree cut method. This study aims to set soft-thresholding power to 4 (scale-free *R* ^2^ = 0.93). Each module contains at least 30 genes, and Pearson correlation analysis was conducted to identify the module with the strongest association with metastasis CRC and examine the relationship among gene modules.

### Differentially expressed genes analysis and enrichment analysis

DEGs in CRC and normal tissue in GSE87211 were screened by the “limma” package in R, with an adjusted *p*-value < 0.05 and |log_2_FC| > 1 considered statistically significant. Gene ontology (GO) and Kyoto encyclopedia of genes and genomes (KEGG) enrichment analyses were performed on the overlapping genes of DEGs and metastasis-related modules.

### Nomogram construction

Univariate Cox analysis was performed to determine the association between the expression of metastasis-related DEGs and patients’ recurrence-free survival (RFS). Lasso penalized Cox regression analysis was used to select metastasis-related genes associated with prognosis. Based on prognostic importance, MXRA8 was identified as an important prognostic molecule, so MXRA8 expression and relevant clinical parameters were used to construct a nomogram. Calibration curves and a receiver operating characteristic (ROC) curve were used to estimate the accuracy and efficiency of the nomogram in a time‐dependent manner.

### Gene set variation analysis and gene set enrichment analysis

GSE39582 and TGA datasets were divided into high and low groups according to the median MXRA8 expression level. Hallmark gene sets were used as a reference gene set. The GSVA package in R was used for GSVA analysis of MXRA8 high and low groups to identify common activation/inhibition pathways. All samples in GSE39582 were divided into two groups according to their risk score. GSEA was conducted to analyze the difference between groups using an adjusted *p*-value < 0.05 and a false discovery rate < 0.25.

### Immune cell infiltration

The enrichment levels of 64 immune signatures in tumor tissues were evaluated by xCell algorithm in GSE87211 dataset. The relative proportions of 22 immune cell types in tumor tissues were evaluated by CIBERSORT algorithm in GSE87211 dataset (16). Correlation analysis of MXRA8 expression levels and immune cells was performed using the Pearson correlation coefficient.

### Plasmid and siRNA

Plasmids overexpressing MXRA8 and an empty vector were purchased from Qinda (Wuhan, China). MXRA8 siRNA and negative siRNA controls were constructed by Qinda (Wuhan, China). The target sequences for MXRA8 siRNAs were AGGACATCCAGCTAGATTA (MXRA8 si1) and CGGGAAAGTCAAAGGGGAA (MXRA8 si2). CRC cells (SW48 and LoVo, purchased from ATCC) were transfected with siRNA or plasmid using Lipofectamine 3000 reagent (Invitrogen, MA, USA) according to manufacturer’s instructions. The knockdown efficiency was validated by qRT-PCR and western blot.

### Cell migration assay

Cell migration was measured using transwell chambers (Beaverbio, Jiangsu, China). Suspensions of 10 × 10^4^ cells in 200 μL of serum-free medium were added to the upper chamber, and a medium containing 10% FBS was added to the lower chamber. After culturing for 12 h, the migrating cells were fixed with 4% paraformaldehyde and stained with crystal violet. The cells were counted in four random fields under a light microscope. The control group was used as the standard and the statistical results of the treatment group were standardized.

### Wound-healing assay

The cells were seeded in 6-well plates and grown to 90% confluence in a complete medium. The artificial wound was made by scraping the confluent cell monolayer with a 200-µL pipette tip, then washed with PBS to remove the detached cells. The remaining cells were grown in a serum-free medium, and cell migration was observed by microscopy and analyzed objectively using Image J. Wound closure (%) was calculated using the following formula: (1−[72-hour area/0-hour area]) × 100.

### Quantitative real-time PCR

Total RNA from cultured cells was extracted using a Trizol reagent kit (Takara, Dalian, China), and qRT-PCR was performed as described previously (17). GAPDH was used as an internal control. The primer sequences were as follows: 5’-GCGGAGGCTACGAATACTCG-3’ (forward), 5’-TCTAGGTCGATGTACTTGGCAG-3’ (reverse), GAPDH: 5’-GGAGCGAGATCCCTCCAAAAT-3’ (forward), 5’-GGCTGTTGTCATACTTCTCATGG-3’ (reverse).

### Western blot

CRC cells transfected with siRNA were collected for protein extraction by using a RIPA buffer (Sigma-Aldrich, Darmstadt, Germany) containing proteinase and phosphatase inhibitors on ice. With the protein concentration being determined, the collected proteins were separated by SDS-PAGE and transferred onto a nitrocellulose membrane (Bio-Rad, Richmond, CA, USA). Post milk blocking, the membranes were incubated with specific primary antibodies (Abcam, ab185444), secondary antibodies and ECL detection reagents (Millipore, USA), for the visualization by chemiluminescence system (UVP, San Gabriel, CA). Image J software was used for protein band quantification.

### Immunohistochemistry

Paraffin-embedded specimens were prepared from tissue samples (35 CRC tissues and 35 paired adjacent normal tissues) collected from 35 patients who had been diagnosed with CRC at the Union Hospital (Wuhan, China) according to the original histopathological reports (**Supplementary Table 1**). All samples were collected with the informed consent of patients. All tissue specimens were collected immediately after surgical excision and quickly fixed in 4% paraformaldehyde solution for 24h. The tissues removed from the fixative were then dehydrated, transparent, waxed, and embedded. The paraffin section was 3μm thick. IHC analysis of tissue was performed using anti-MXRA8 (Abcam, ab185444, 1: 100) and anti-CD8 (Abcam, ab209775, 1:2000 dilution) antibodies and overnight incubation at 4°C. After epitope retrieval, H_2_O_2_ treatment and non-specific antigens blocking, chips were next incubated with secondary antibody as described previously (18). The IHC results were scored by two independent observers.

### Statistical analysis

All statistical analyses were performed using R software 4.0.3. The Student’s *t*-test was used to determine the significance of DEGs, the cell migration assay, and the wound-healing assay. The Wilcoxon test was applied to determine the significance of the difference between the risk score and clinicopathological characteristics. GraphPad Prism 8.00 software was used to calculate the area under the curve. ****p<0.0001; ***p<0.001; **p<0.01; *p<0.05; ns, not significant.

## Results

### The turquoise/yellow module was identified as the pivotal module associated with metastasis and CD8^+^ by WGCNA

**Figure 1:**
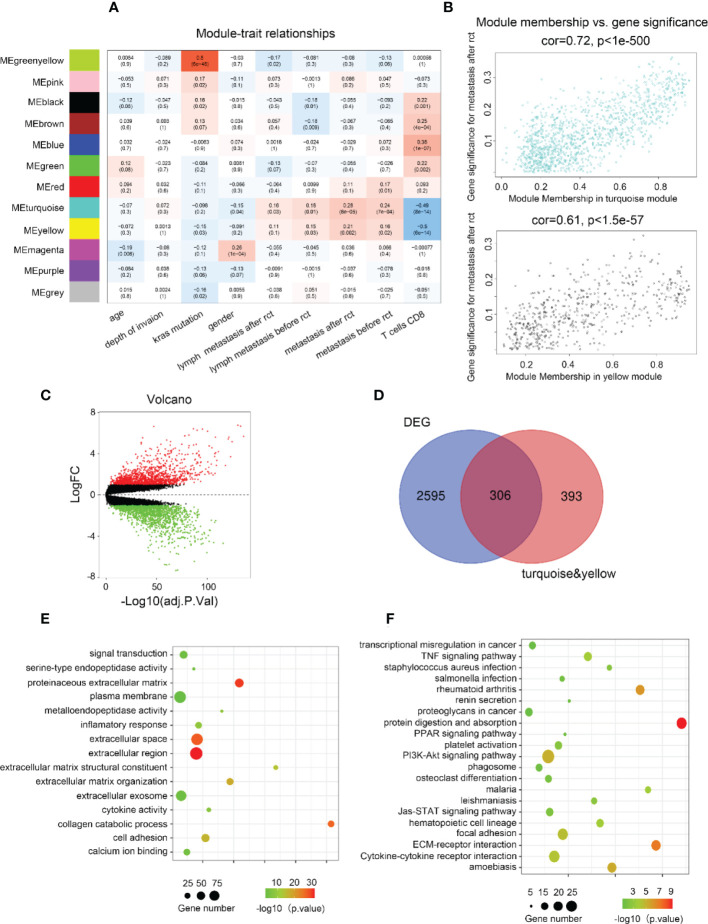
Biological function and pathway annotation. **(A)** Heatmap of the correlation between modules and cancer hallmarks. **(B)** Correlation between turquoise/yellow module and metastasis. **(C)** The volcano plot of differentially expressed genes (DEGs) between colorectal carcinoma samples and normal colorectal tissue samples (logFC > 2 and adjusted p-value <0.05). The horizontal axis represents the adjusted p-value, and the vertical axis represents the fold change. Red and green circles indicate up- and down-regulated genes, respectively. **(D)** Venn plot of the intersection of upregulated differentially expressed genes and selected genes from WGCNA. **(E)** The top 15 GO functions enriched for the upregulated 306 genes. **(F)** The top of 21 KEGG pathways enriched for the up-related 306 genes.

Considering the significance of metastasis in determining the prognosis of CRC patients, WGCNA was used to analyze the co-expression patterns between metastasis and whole-transcriptome profiling data (**Figure S1A**). The optimal soft threshold was set to 4 to construct a scale-free network (**Figure S1B**) to identify 12 modules (**Figures 1A**, **S1C**). The turquoise and yellow module highly correlated with metastasis were chosen for further analysis (**Figure 1B**). Gene expression profiles from GSE87211 identified 2901 upregulated DEGs in CRC samples compared to normal control tissues (**Figure 1C**). In current study, 699 genes with the highest connectivity in the turquoise/yellow module were intersected with the 2901 DEGs, outputting 306 candidate genes (**Figure 1D**). GO functions and KEGG pathways enriched analysis indicated that the genes were related to metastasis functions (**Figures 1E, F**).

### MXRA8 was selected as a hub gene associated with metastasis

**Figure 2:**
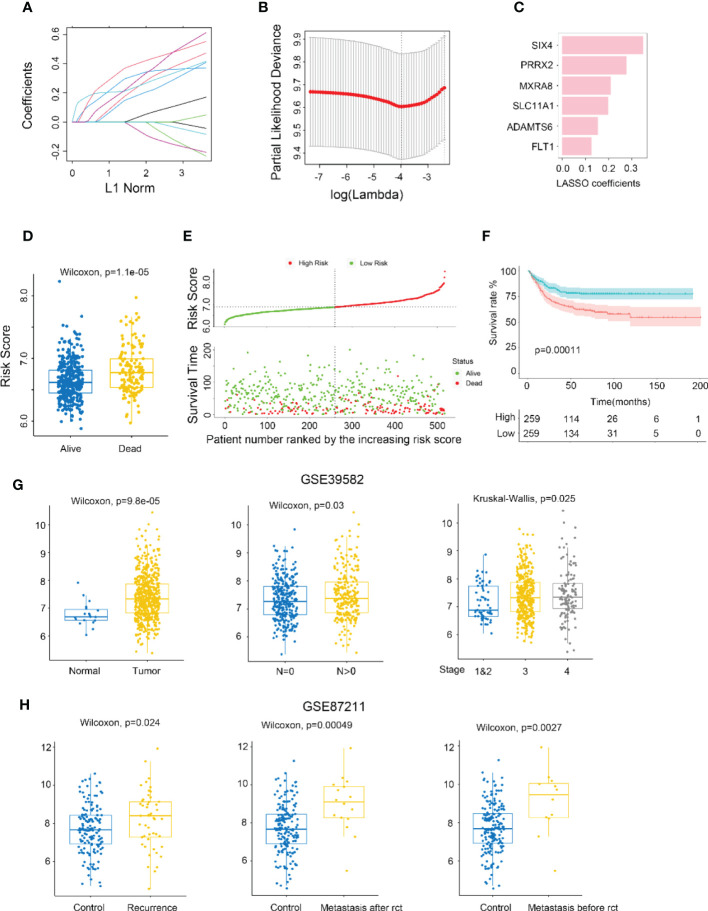
Identifying MXRA8 as a hub gene. **(A)** LASSO coefficient profiles of metastasis-related prognostic differential expressed genes. **(B)** 10-fold cross-validation for penalty parameter λ selection in LASSO model. **(C)** LASSO coefficients of six metastasis-related genes. **(D)** Comparison of risk scores in alive and dead patients. **(E)** The distribution of risk score, patients’ status, and RFS time. **(F)** Kaplan–Meier RFS curves for patients in high- and low-risk groups. **(G)** Boxplot indicating MXRA8 expression in normal/tumor (left), lymph metastasis or not (middle), and different stages (right) from GSE39582 database. **(H)** Boxplot indicating MXR8 expression in normal/recurrence (left), normal/metastasis after resection (middle), normal/metastasis before resection (right) from TCGA database.

The metastasis-related gene signature (MGS) was constructed by using LASSO Cox regression analysis to screen the most significant prognostic markers within the module (**Figures 2A-C**), consisting of six genes (SIX4, PRRX2, MXRA8, SLC11A1, ADAMTS6, and FLT1). The MGS score of each patient was calculated based on the expression levels of the six genes. The median MGS score was regarded as the cutoff, with all patients being classified as MGS-high or MGS-low, and dead CRC patients having a higher risk score than live patients (**Figure 2D**). A shorter survival time was found in CRC patients with MGS-high by survival analysis (**Figure 2E**), which was consistent with the Kaplan-Meier analysis results (*p*<0.0001) (**Figure 2F**).

TNM stage and risk score were independent risk factors for RFS by Multivariate Cox regression analysis (**Figures S2A, B**). The expression of risk score-high group genes was related with metastasis pathways (EMT, angiogenesis, hedgehog signaling, and notch signaling pathway) (*p*< 0.0001) by GSEA (**Figure S2C**). A nomogram for forecasting the CRC patients’ survival probability was established by combining the risk score and clinicopathological characteristics (age, sex, and stage) of the patients (**Figure S3A**). The probabilities for 3-, 5-, and 10-year survival predicted by the nomogram highly accorded with the observed values (**Figure S3B**). The area under the ROC curves for 3-, 5-, and 10-year OS were 0.700, 0.692, and 0.763, respectively (**Figure S3C**). Moreover, the AUC values presented that the risk score combined with tumor stage showed the best ability to predict OS among the factors analyzed (**Figure S3D**).

MXRA8 has been scarcely any report in most cancers, but being of great importance for CRC prognostic (**Figure 2C**). Higher expression of MXRA8 was found in tumors (compared to normal), in CRC patients with positive lymphatic metastasis (compared to negative lymphatic metastasis), in CRC patients with more advanced stage (**Figures 2G**; **S3E, F**), and patients with recurrence and metastasis (compared to no recurrence and metastasis) (**Figure 2H**).

### Construction of an MXRA8-based prognostic prediction model

**Figure 3:**
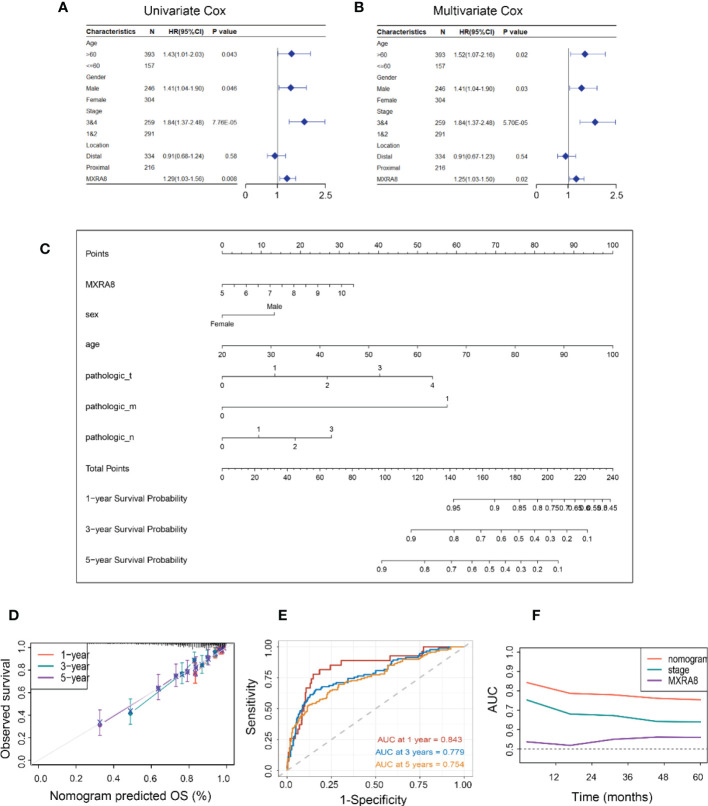
Constructing an MXRA8-based prognostic prediction model. **(A)** Univariate Cox regression analysis of MXRA8 and clinicopathological characteristics **(B)** Multivariate Cox regression analysis of MXRA8 and clinicopathological characteristics. **(C)** Nomogram developed based on MXRA8 and clinicopathological characteristics. **(D)** Plots depict the calibration of the model regarding the agreement between predicted and observed OS. Model performance is shown by the plot relative to the 45-degree line, representing perfect prediction. Calibration analysis of the agreement between nomogram predicted 1-, 3-, and 5-year survival and observed outcomes. **(E)** Time-dependent ROC curves at 1, 3, and 5 years of the nomogram. **(F)** AUC plotted for different durations of OS for nomogram-based signature, tumor stage, and MXRA8 in TCGA datasets.

MXRA8 expression was statistically significant by univariate Cox regression analysis (**Figure 3A**) and identified as an independent prognostic biomarker in the multivariate Cox proportional hazards regression model using GSE39582 data (HR = 1.25, 95% confidence interval (CI) = 1.03–1.50, *p*= 0.02, **Figure 3B**). A nomogram for forecasting the CRC patients’ survival probability was established by combining MXRA8 and clinicopathological characteristics (age, sex, and stage) (**Figure 3C**). The probabilities for 1-, 3-, and 5-year survival predicted by the nomogram highly accorded with the observed values (**Figure 3D**). The area under the ROC curves for 3-, 5-, and 10-year OS were 0.843, 0.779, and 0.754, respectively (**Figure 3E**). Moreover, the AUC values presented that MXRA8 combined with tumor stage showed the best ability to predict OS among the factors analyzed (**Figure 3F**).

### MXRA8 is involved in cancer-related signaling pathways in CRC

**Figure 4:**
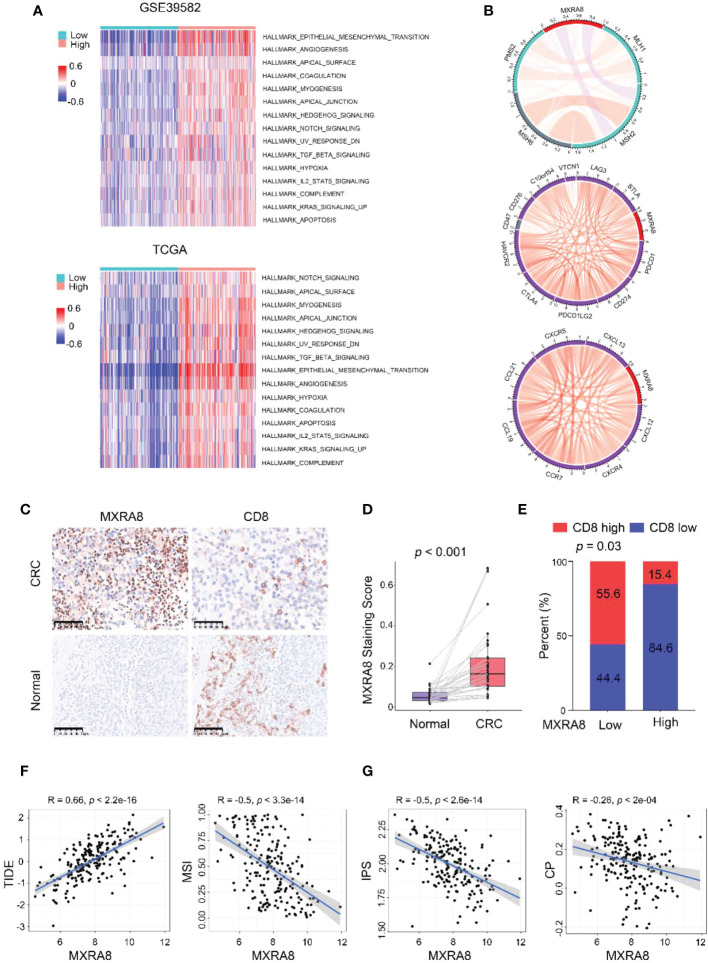
MXRA8 promotes CRC migration and immunosuppression. **(A)** GSVA analysis of hallmark and KEGG pathway gene sets in MXRA8 high and low expression samples from GSE39582 and TCGA datasets. **(B)** In TCGA, MXRA8 expression is negatively associated with microsatellite instability but positively associated with immune checkpoint molecule expression, chemokines, and chemokine receptors expression. **(C)** IHC images of MXRA8 protein expression in normal and tumor tissue. Scale bars, 50 μm. **(D)** Statistical analysis of MXRA8 expression in CRC and adjacent normal tissues. **(E)** Statistical analysis of the correlation between MXRA8 and CD8 expression in CRC tissues. **(F, G)** MXRA8 expression is positively linked to tumor immune dysfunction and exclusion (TIDE) and negatively associated with microsatellite instability (MSI), immunophenoscore (IPS), and checkpoint (CP).

KEGG pathway gene sets and GSVA analysis of hallmark in MXRA8 high and low expression samples from GSE39582 and TCGA datasets revealed that tumor metastasis-related pathways enrichment in the MXRA8 high group (**Figure 4A**). MXRA8 was highly negatively associated with microsatellite instability but positively associated with immune checkpoint molecule expression, chemokines, and chemokine receptor expression (**Figure 4B**). In IHC staining, MXRA8 protein expression increased in tumor tissue (**Figures 4C, D** and **S4A**). Furthermore, MXRA8 and CD8 negatively correlated with CRC expression (**Figure 4E**). MXRA8 expression is positively associated with Tumor Immune Dysfunction and Exclusion (TIDE) and negatively associated with microsatellite instability (MSI), the immunophenoscore (IPS), and checkpoint (CP) (**Figures 4F, G** and **S4B**).

### MXRA8 promotes CRC cell invasion and migration *in vitro*

**Figure 5:**
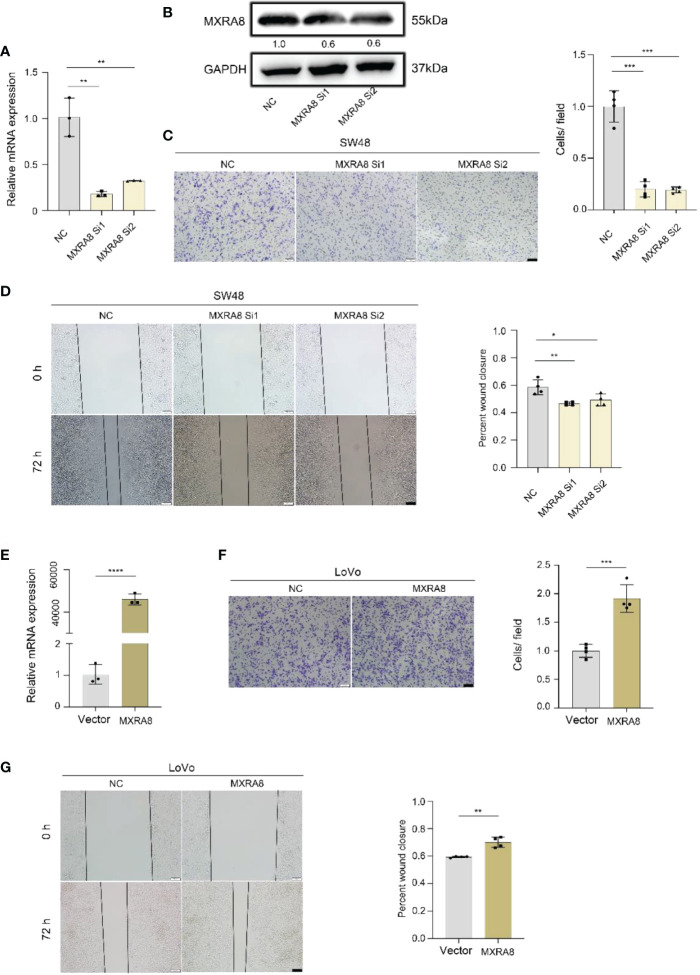
MXRA8 promotes CRC cell invasion and migration *in vitro*. **(A-D)** Levels of MXRA8 mRNA, MXRA8 protein, images of transwell assay for migration, and wound healing assay in SW48 transfected with MXRA8 siRNA. **(E-G)** Levels of MXRA8 mRNA, images of transwell assay for migration, and wound healing assay in LoVo with MXRA8 plasmid transfection. ****p<0.0001; ***p<0.001; **p<0.01; *p<0.05.

*In vitro*, the ability of invasion and migration was assessed by MXRA8 knockdown in SW48 or plasmid transfection in LoVo (**Figures 5A, E**). The protein expression of MXRA8 in SW48 cells was decreased followed by MXRA8 knockdown (**Figure 5B**). The migratory and invasive abilities were reduced with MXRA8 knockdown by transwell assays in SW48 (**Figure 5C**). Cell migratory ability was repressed with MXRA8 knockdown by wound-healing assays in SW48 (**Figure 5D**). Furthermore, transwell assays showed that the invasive and migratory abilities were enhanced with MXRA8 plasmid transfection in LoVo (**Figure 5F**). A wound-healing assay illustrated that the cell migratory ability was upregulated when MXRA8 was overexpressed in LoVo (**Figure 5G**). These results corroborated that MXRA8 played a pivotal role in CRC migration and invasion *in vitro*.

### High expression of MXRA8 correlates with low CD8^+^ T cell infiltration

**Figure 6:**
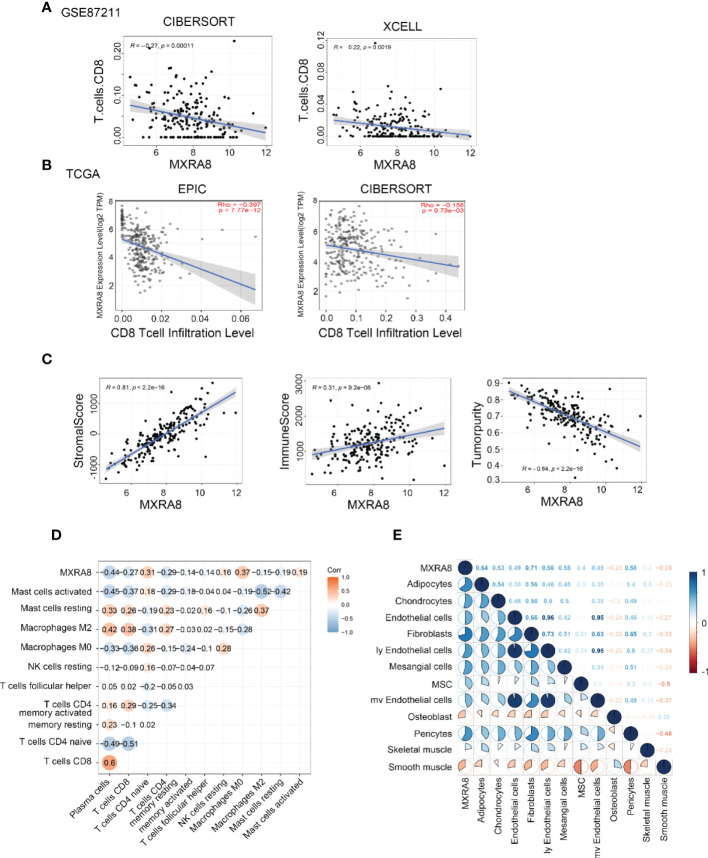
High MXRA8 expression correlates with low CD8^+^ T cell infiltration level. **(A)** The protein expression of MXRA8 is negatively correlated with CD8^+^ T cell infiltration level in GSE87211 with CIBERSORT and XCELL algorism. **(B)** The mRNA expression of MXRA8 is negatively correlated with CD8^+^ T cell infiltration level in TCGA with EPIC and CIBERSORT algorism. **(C)** The protein expression of MXRA8 is positively correlated with a stromal score and immune score but negatively associated with tumor purity. **(D)** Correlation analysis between MXRA8 expression levels and immune cells **(E)** Correlation analysis between MXRA8 expression levels and stromal cells infiltration.

Several algorithms were used to conduct the following study in CRC, and the expression of MXRA8 was negatively correlated with CD8^+^ T cell levels in GSE87211 and TCGA datasets (**Figures 6A, B**). The protein expression of MXRA8 is negatively correlated with the stromal and immune scores but positively with tumor purity (**Figure 6C**). A correlation matrix between MXRA8 and immune cells/stromal cells revealed that MXRA8 is negatively correlated with CD8^+^ T cells but positively with multiple types of stromal cells (skeletal muscle, pericytes, mv endothelial cells, ly endothelial cells, fibroblasts, endothelial cells, chondrocytes, and adipocytes), suggesting its potential role meditated by CD8^+^ and the stromal cells in tumor progression (**Figures 6D, E**). The prognostic value of MXRA8 was validated using TCGA cohorts. In the univariate Cox regression analysis, MXRA8 expression was statistically significant (left of **Figure S5A**). Furthermore, multivariate Cox regression analysis indicated that MXRA8 was an independent risk factor for overall survival in CRC (HR = 1.61, 95% CI = 1.01–2.67, right of **Figure S5A**). Furthermore, higher expression of MXRA8 was associated with a poorer survival rate (**Figures S5B-D**), indicating that high MXRA8 expression is an unfavorable prognostic biomarker for CRC (**Figure S5**).

## Discussion

Tumor metastasis and CD8^+^ T cell infiltration play a crucial role in CRC patient survival. In this study, we conducted WGCNA to explore vital modules of tumor metastasis and CD8^+^ T cell infiltration, then with hub gene selection and survival analysis. A CRC prognosis prediction model based on six related genes was constructed, of which one gene, MXRA8, shows potential as a biomarker for survival and CD8^+^ T cell infiltration in CRC.

The comprehensive evaluation of MXRA8 in four independent CRC cohorts demonstrated high expression of MXRA8 in tumors (compared to normal), in CRC patients with positive lymphatic metastasis (compared to negative lymphatic metastasis), advanced stage, recurrence, and metastasis. The nomogram, including MXRA8 and tumor stage, also showed good prognostic, predictive performance. To further clarify the role of MXRA8 in cancer, we conducted GSVA analysis of hallmark and KEGG pathways, showing that high expression of MXRA8 was positively associated with migration and immunosuppression.

MXRA8 is highly expressed in CRC and associated with CRC metastasis. MXRA8 is a transmembrane protein that can influence integrin signaling and regulate cell-cell interactions (19, 20). MXRA8 also serves as a receptor for multiple arthritogenic alphaviruses (21). The function of MXRA8 in cancer development and progression has not been addressed, but it has been reported to be highly expressed in thyroid cancer (22), kidney cancer (15), esophageal cancer (23), and pancreatic cancer (24), Therefore, this study is the first report on the high expression of MXRA8 in CRC. Additionally, MXRA8 is associated with CRC metastasis, and increased MXRA8 promotes CRC invasion and metastasis *in vitro*. This is similar to the results of a recent study by Roger et al., which found that MXRA8 is highly expressed in lung metastasis of breast cancer, and miR-200s can down-regulate MXRA8 expression to inhibit the growth and metastasis of breast tumor cells *in vivo* (25).

MXRA8 promotes CRC invasion and metastasis through multiple mechanisms and is involved in tumor invasion and metastasis. EMT-like changes in tumor cells not only loosen cell-cell adhesion complexes, enhancing cell migration and invasive properties but are also associated with enhanced stem cell properties and drug resistance (26, 27). The present study depicted that the MXRA8 high expression group revealed significant enrichment of EMT and angiogenesis. MXRA8 was confirmed to be an adhesion molecular protein expressed in epithelial and mesenchymal cells (28). These results suggest that MXRA8 may be involved in cell adhesion and migration. Hypoxia and TGF-β signaling can promote tumor EMT and angiogenesis in multiple ways and are thought to contribute to tumor invasion and metastasis (29, 30). In our study, the high MXRA8 group showed significant enrichment of hypoxia and TGF-β signaling pathways. In addition, our study revealed that MXRA8 expression correlated with the expression of multiple metastasis-associated chemokines (CXCL12, CXCL13, CCL9, CCL21, CXCR4, CXCR5, and CCR7) (31–37), suggesting that MXRA8 may be involved in tumor invasion and metastasis by regulating the secretion of chemokines. Numerous studies have reported that chemokines can regulate tumor invasiveness and metastasis and play a crucial role in establishing the composition of the “pre-metastatic niche” (38). For example, the CXCL12/CXCR4 axis is involved in tumor growth, invasion, angiogenesis, and metastasis in CRC, breast and pancreatic cancers (39–42). Nonetheless, the role of MXRA8 in tumor metastasis still requires further study.

MXRA8 levels are associated with cancer immunity, and ICI is changing the treatment paradigm for many cancers (43), with adequate infiltration of tumor-reactive CD8^+^ T cells a prerequisite for the ICI response (44). In colorectal cancer, IL-2 activates TPH1-5-HTP-AhR signaling in the tumor microenvironment to induce CD8^+^ T cell exhaustion in tumor tissues (45). In addition, significant enrichment of immunosuppressive cytokines TGFB1 and IL10 have been found in the Epithelial-mesenchymal transition-high group of almost all cancer types, forming an immunosuppressive microenvironment and leading to decreased infiltration of CD8^+^ T cells (46). Inefficient antigen presentation due to immune escape is also an important factor leading to poor infiltration of CD8^+^ T cells. T cell suppressor receptors such as CTLA-4, PD-1, and TIGIT are essential for T cell activation, antigen recognition, and recruitment, and can inhibit effective anti-tumor immune responses (47). These signaling pathways (TGF-β, EMT, Hypoxia), participating in limiting CD8^+^ T cell infiltration, have been preliminary demonstrated to be associated with high expression of MXRA8 in this work. Furthermore, MXRA8 was linked to the expression of several immune checkpoints in our work, including PD-1, PD-L1, PD-L2, CTLA-4, TIM-3, and LAG-3; thus, MXRA8 may be involved in tumor immune escape. While more in-depth studies between MXRA8 and CD8^+^ T cell infiltration are needed, we propose some ideas about it.

MXRA8 mRNA levels were inversely related to the abundance of most of the immune cell types, especially plasma cells, M2 macrophages, and CD4 memory cells. Correlation analysis showed that the expression of MXRA8 correlated with the expression of many stromal cells, including endothelial cells, fibroblasts, and adipocytes. In many previous studies, fibroblasts and endothelial cells play key roles in cancer progression by promoting extracellular matrix deposition and remodeling, EMT, invasion, metastasis, and therapy resistance (48, 49). These results demonstrate that MXRA8 may affect the development and prognosis of cancers by shaping the tumor microenvironment.

TIDE was recently evaluated as a potential biomarker to predict the response to ICI therapy in prospective clinical trials and many tumor types (50). TIDE prediction scores correlated with T cell dysfunction in Cytotoxic T Lymphocyte (CTL)-high tumors and T cell exclusion in CTL-low tumors (51). In our study, patients with high MXRA8 expression had less CTL infiltration and higher TIDE and T cell exclusion scores so that MXRA8 may be involved in tumor immune escape through T cell exclusion. The IPS function was used to measure the immune state of the samples (52), and the higher the composite score of IPS, the stronger the immunogenicity of the sample. Our study showed that patients with high MXRA8 expression had lower IPS scores and antigen immunogenicity, indicating poor responsiveness to immunotherapy, which is consistent with the TIDE predictions. Overall, these results suggest that patients with low MXRA8 expression may have a better response to immunotherapy and that MXRA8 may be a potential biomarker for predicting the efficacy of CRC immunotherapy.

## Conclusion

This study first found that MXRA8 was overexpressed in CRC. Meanwhile, MXRA8 expression was strongly correlated with tumor malignancy, metastasis, recurrence, and immunosuppressive microenvironment. Furthermore, MXRA8 expression predicts poor prognosis and is an independent prognostic factor for OS in CRC. MXRA8 can also serve as a potential biomarker for immunotherapy. In the future, the role of MXRA8 in CRC prognosis and immunotherapy should be validated in prospective, multicenter, and randomized clinical trials that include follow-up data and receive immunotherapy.

## Data availability statement

The datasets presented in this study can be found in online repositories. The names of the repository/repositories and accession number(s) can be found in the article/**Supplementary Material**.

## Author contributions

LT: Data curation, Formal analysis, Methodology, Software, Validation, Visualization, Writing - original draft. DF: Methodology, Investigation, Validation, Software, Formal analysis. FL: Methodology, Validation, Writing - review & editing. JL: Validation, Writing - review & editing. YZ: Funding acquisition, Writing - review & editing. JG: Methodology, Resources, Supervision. KT: Resources, Supervision. GW: Resources, Supervision. LW: Resources, Writing - review & editing, Supervision. ZW: Conceptualization, Writing - review & editing, Supervision, Funding acquisition. All authors contributed to the article and approved the submitted version.
