# Supplementary material for: MXRA8 is an immune-relative prognostic biomarker associated with metastasis and CD8+ T cell infiltration in colorectal cancer
Source: Front Oncol. 2023 Jan 10;12:1094612. doi: 10.3389/fonc.2022.1094612 (PMC9871988; doi:10.3389/fonc.2022.1094612)
Supplement: Supplementary file 1 [file DataSheet_1.docx]

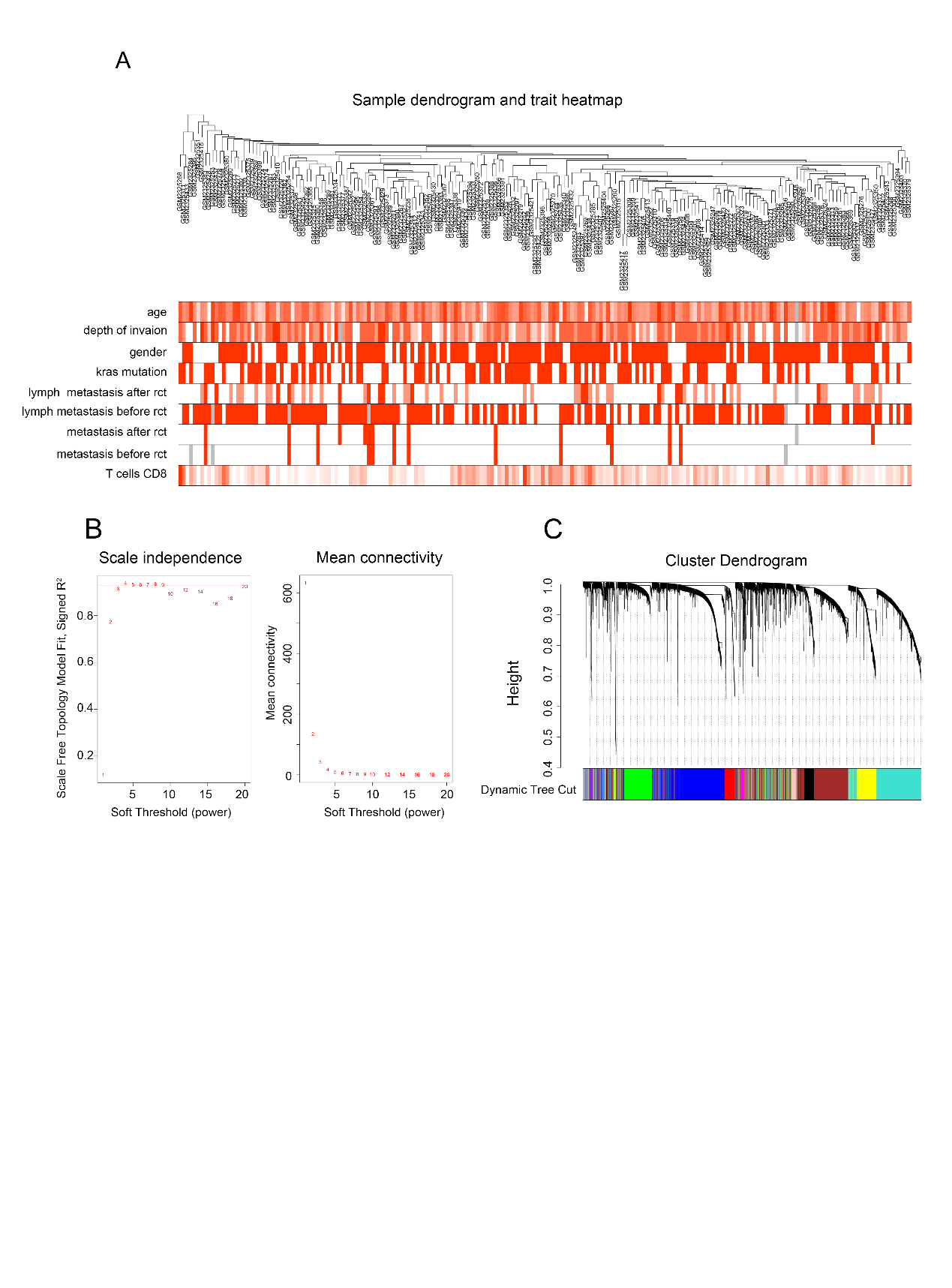


**Figure S1** | WGCNA construction and gene significance for metastasis after resection. (A) Clustering dendrogram of 203 samples from WGCNA. (B) Calculating the scale-free index for different soft-threshold powers (β) and the mean connectivity for different soft-threshold powers. (C) Clustered dendrogram of differentially expressed genes.


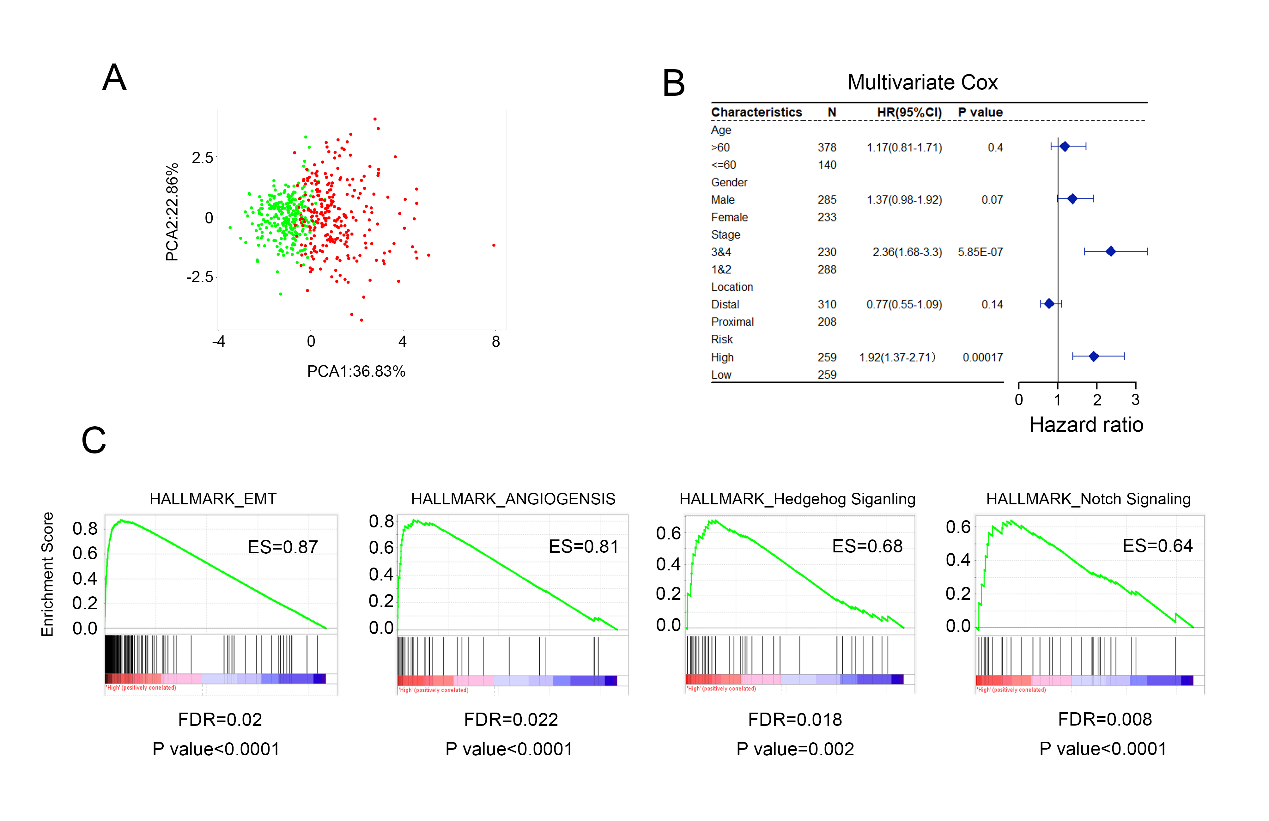
**Figure S2** | High MXRA8 expression correlates with metastasis. (A) Principal component analysis (PCA). (B) Multivariate Cox regression analysis of risk and clinicopathological characteristics. (C) GSEA of EMT, angiogenesis, hedgehog signaling, and notch signaling pathway in high-risk groups.


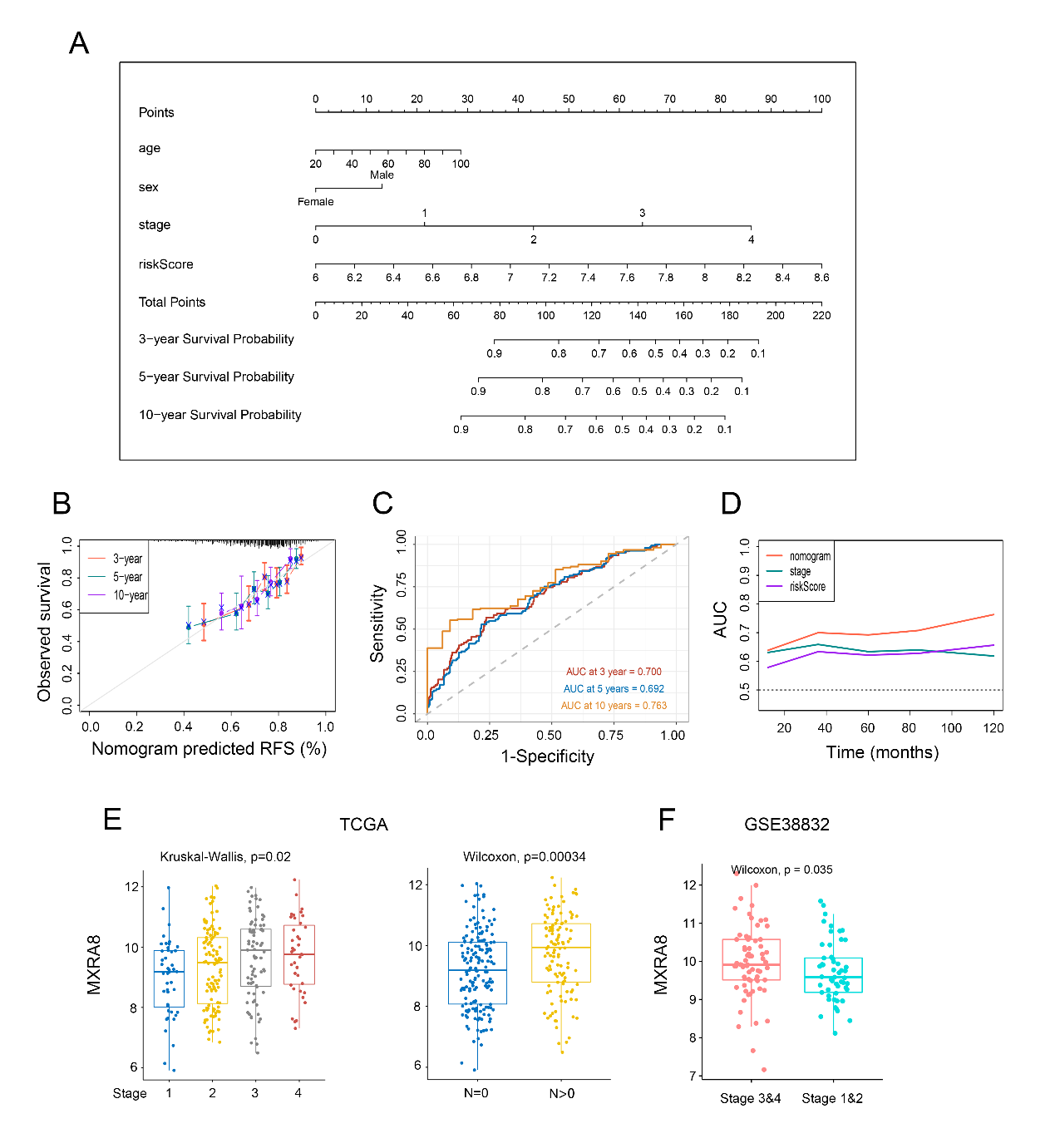


**Figure S3** | Constructing a prognostic prediction model based on six genes (SIX4, PRRX2, MXRA8, SLC11A1, ADAMTS6, and FLT1). (A) Nomogram developed based on risk score and clinicopathological characteristics. (B) Plots depict the calibration of the model regarding the agreement between predicted and observed OS. Model performance is displayed by the plot relative to the 45-degree line, representing perfect prediction. Calibration analysis of the agreement between nomogram predicted 3-, 5-, and 10-year survival and observed outcomes. (C) Time-dependent ROC curves at 3, 5, and 10 years of the nomogram. (D) AUC plotted for different durations of OS for nomogram-based signature, tumor stage, and risk score in TCGA datasets. (E) Boxplot indicating MXRA8 expression in different stages (left) and lymph metastasis (right) from TCGA database. (F) Boxplot indicating MXRA8 expression in different stages from GSE38832.


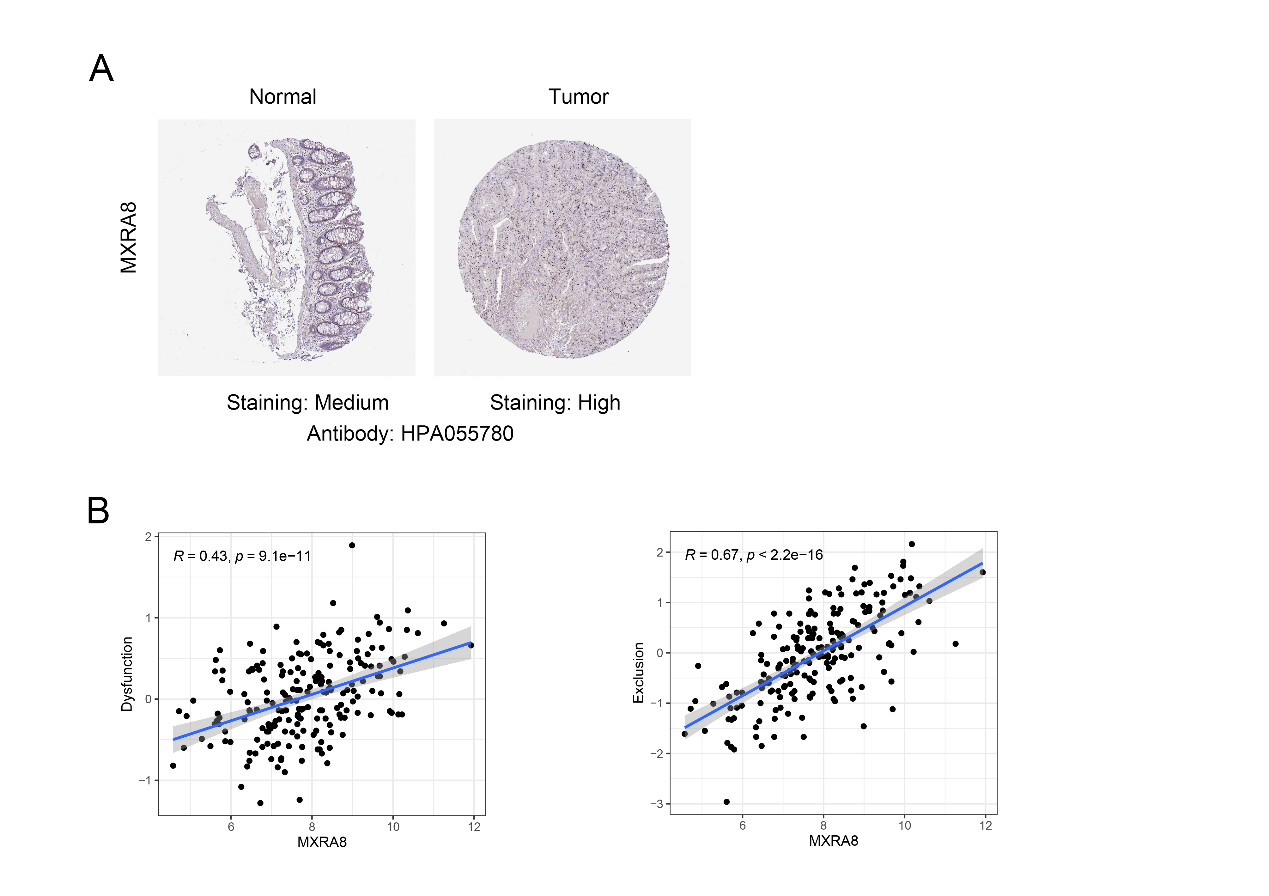


**Figure S4** | MXRA8 protein expression and correlation with T cell. (A) IHC images of MXRA8 protein expression in human protein atlas database. (B) The correlation between MXRA8, T cell exclusion score, and T cell dysfunction score.


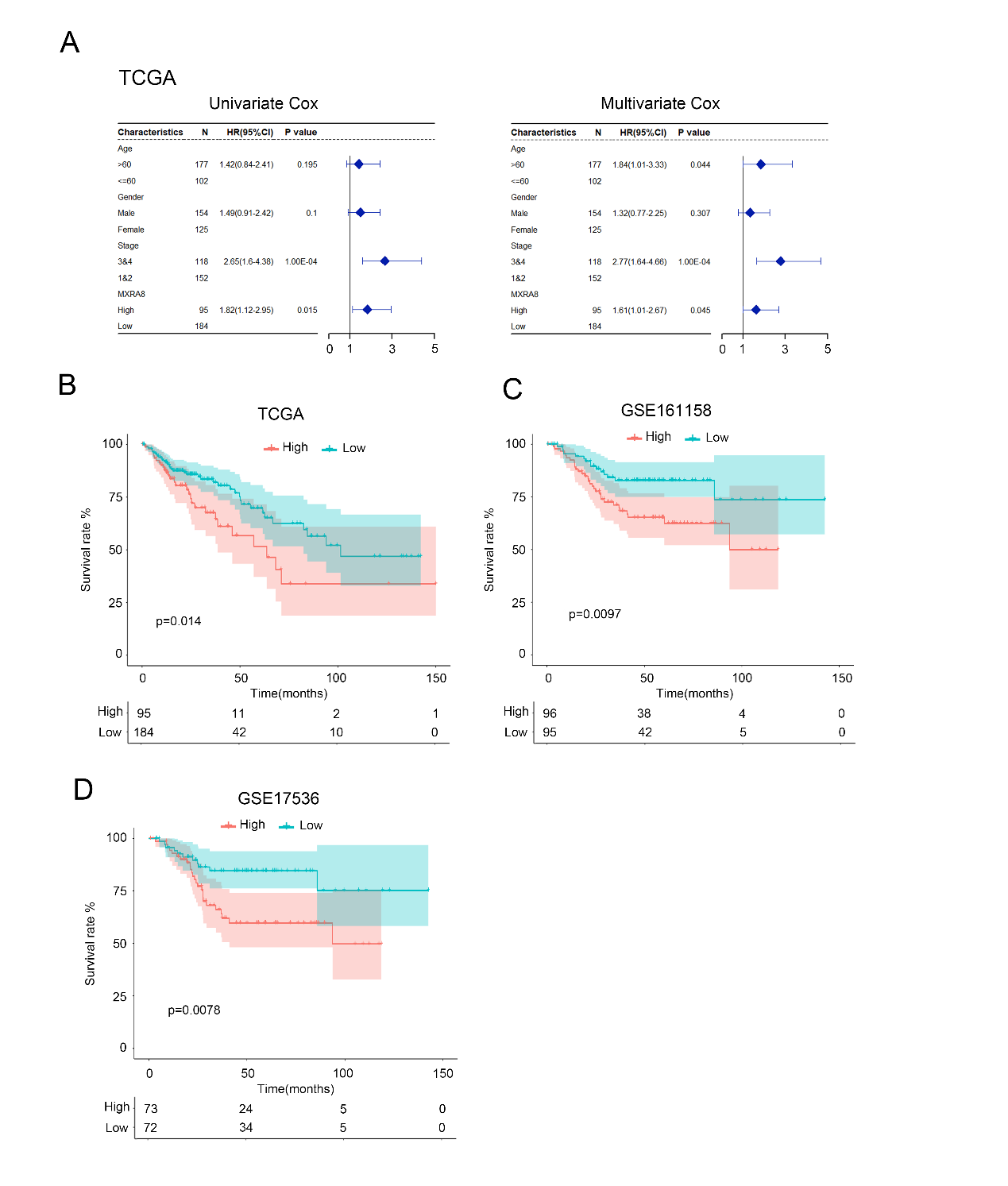


**Figure S5 |** MXRA8 expression could predict the clinical benefit. (A) Univariate and multivariate Cox regression analyses of MXRA8 and clinicopathological characteristics in TCGA. (B) Kaplan–Meier OS curves for patients in high- and low-risk groups in TCGA. (C) Kaplan–Meier OS curves for patients in high- and low-risk groups in GSE16158. (D) Kaplan–Meier OS curves for patients in high- and low-risk groups in GSE17536.

Supplementary Table 1: Clinical data of 35 CRC patients.

| Clinical features | N |
| --- | --- |
| All patients | 35 |
| Age |  |
| >=65 | 18 |
| <65 | 17 |
| Gender |  |
| Male | 24 |
| Female | 11 |
| Stage |  |
| Ⅰ&Ⅱ | 21 |
| Ⅲ&Ⅳ | 14 |
